# Evaluation of Shallow Groundwater Quality at Regional Scales Using Adaptive Water Quality Indices

**DOI:** 10.3390/ijerph191710637

**Published:** 2022-08-26

**Authors:** Petre Bretcan, Danut Tanislav, Cristiana Radulescu, Gheorghe Serban, Serban Danielescu, Michael Reid, Daniel Dunea

**Affiliations:** 1 Faculty of Humanities, Valahia University of Târgovişte, 130105 Târgovişte, Romania; 2 Faculty of Sciences and Arts, Valahia University of Târgovişte, 130004 Târgovişte, Romania; 3 Faculty of Geography, Babes-Bolyai University, 400084 Cluj-Napoca, Romania; 4 Fredericton Research and Development Centre, Environment and Climate Change Canada and Agriculture and Agri-Food Canada, Fredericton, NB E3B 4Z7, Canada; 5 Department of Geography and Planning, School of Humanities, Arts and Social Studies, University of New England, Armidale, NSW 2351, Australia; 6 Faculty of Environmental Engineering and Food Science, Valahia University of Târgovişte, 130004 Târgovişte, Romania

**Keywords:** shallow groundwater, WQI, IwWQI, entropy, hot-spot analysis, total hazard index, cumulative carcinogenic risk

## Abstract

Groundwater, which is the main source of water for human consumption in many rural areas, has its quality determined by the complex interaction of environmental factors and anthropogenic activities. The present study evaluated the quality of shallow groundwater (1 to 25 m depth) in the rural area of the Târgovişte Plain, a densely populated area (200 inhabitants/km^2^) using 80 water samples collected from public wells. In order to explain the spatial distribution of the concentrations of the 19 physicochemical parameters considered (including heavy metals), the evaluation of groundwater quality for human consumption and potential impact on human health was conducted using the Water Quality Index (WQI), Integrated Weight Water Quality Index (IwWQI), Total Hazard Index (THI), and cumulative carcinogenic risk (CCR). For the WQI/IwWQI the comparative analysis of the two indices showed that for the WQI, it is important to select an optimal set of parameters, because use of a large number of physicochemical parameters can eclipse the values that exceed WHO guideline limits. In contrast, the use of entropy in the calculation of the IwWQI did not lead to eclipsing of exceedance, no matter the number of parameters used. Areas with poor and very poor groundwater quality according to the WQI/IwWQI overlapped, with a moderate risk to human health (THI > 1) for noncarcinogenic contaminants and also a risk of developing cancer according to the CCR average value (1.15 × 10^−2^). The health of 43% of the rural population in the Târgovişte Plain can be affected if they drink contaminated groundwater, and it is estimated that about 600 people can develop cancer during their lifetime. If the risk of developing cancer is reduced only in the rural population that does not have access to a water source from a centralized and verified network, the results suggest that 385 people (1.15%) can develop cancer as a result of consuming groundwater contaminated with heavy metals based on the average value of CCR. This value is lower than the general mortality rate in areas with high CCR and below the average number of cancer patients in Romania (2.65%). The quality of groundwater and the risk of developing diseases and cancer due to water consumption is directly proportional to the intensity of agricultural land use and inversely proportional to the depth of the groundwater layer, the distance from the main hydrographic network and the reservoirs, and the distance from the main city, Târgovişte. The complex and integrated analysis of groundwater quality using quality indices and indicators of health risk for the population, validated by hot-spot analysis and compared to the mortality rate, is an approach with practical applicability. This integrated approach allows public authorities, policymakers, and health services to implement an efficient monitoring program and optimize anthropogenic activities in order to prevent groundwater contamination and finally improve the quality of life for the residents in the area of this study.

## 1. Introduction

Groundwater is an important source of water around the world [1,2,3,4], and the quality, quantity, and easy access to this resource have been linked to the evolution or decline of various human communities [5]. In most European countries, 75% of the total water consumed by the population comes from underground sources, in India the percentage for rural populations is similar, at around 80%, while in the USA 45% of land is irrigated from underground sources [6,7]. In rural areas, shallow groundwater use typically includes domestic use, drinking, and irrigation [3,5,8].

From a water resources perspective, groundwater has better quality than surface water in many cases. Shallow groundwater is also generally easily accessible with low operating costs. However, shallow groundwater resources are at risk due to overexploitation and contamination from various sources [9,10,11,12]. The significance of negative impacts on groundwater resources has been linked in the past to the depth of the groundwater bodies, their proximity to contamination sources, and the shape of the respective groundwater bodies [13,14,15,16]. Shallow groundwater resources can benefit from a “natural protection” against contamination due to hydrogeological settings (e.g., low-permeability soils, presence of shallow confining layers). However, this protection is not always sufficient to prevent chemical contaminants from leaching into the water wells used as a source for drinking [17], domestic consumption [18,19,20], or irrigation purposes [21]. Hence, groundwater resources require careful monitoring for prevention or early detection of contamination.

It is challenging to compare water samples that come from multiple sources and locations, particularly when there are multiple parameters of interest considered for assessing the level of contamination and/or potability of the respective waters. Hence, a series of water quality indices were developed in the past to assess the degree of contamination of groundwater bodies. These indices are most useful when dealing with contamination of large groundwater bodies, when the contamination sources are numerous and diverse [22,23], and the number of water quality parameters available (e.g., chemical, physicochemical, and/or biological) is large. One advantage of using indices is that they can target the assessment of the water quality from various perspectives. For example, the quality of a groundwater body can be deemed “poor” from a drinking water perspective, but “appropriate” for industrial or agricultural activities.

Starting with the index created by Horton in 1965 [24], numerous quality indices have been proposed over time. Indices have utilized various methods to select parameters and calculate final indices, such as the linear sum of different subindices [25,26,27], fuzzy logic [28,29], probability theory [30,31,32], or artificial intelligence [33,34].

One of the challenges related to the use of water quality indices is the number of parameters considered, and hence, a key step in developing a water quality index or selecting the water quality index to be used is the selection of an optimal number of parameters to include. This is necessary to avoid “eclipsing”, which can occur when a number of parameters exceed the water quality guidelines [35,36]. At the same time, flexibility in defining the water quality index to be used is required, because over time, the mix of dominant pollutants or pollutants of interest might change [37,38]. For selecting the parameters included in the water quality index, the local hydrogeological conditions, climate, land use, anthropogenic influences, and the use of water resources have to be considered [20,39]. Not including heavy metals or other chemical constituents that present a high risk for human consumption in the final value of the groundwater quality indices can create the false impression that water is “excellent” or “good”, even when the presence of heavy metals would indicate the opposite. The inclusion of heavy metals in the water quality indices is important, as dermal absorption and long-term ingestion of water with high concentrations of heavy metals can result in their accumulation in tissue and adverse effects on the circulatory, nervous and immune systems. Due to their high toxicity, certain heavy metals (Ni, Pb, and Cr) increase the risk of developing various types of cancer [40,41].

In Romania, the systemic and continuous monitoring of groundwater is conducted in accordance with European legislation (e.g., Water Framework Directive 2000 and Groundwater Pollution Protection Directive 2006) and has been designed for assessing the spatial and temporal variability of the analyzed parameters as well as the impact of changing environmental conditions due to both natural and anthropogenic factors.

The aim of this research was to evaluate the quality of groundwater from the rural area of Târgovişte Plain (~1000 km^2^) for human consumption and the health risk for the population in an area with intensive industrial and agricultural activities where groundwater resources are currently under significant stress. This is one of the first studies that involves the use of groundwater quality indices in Romania. The main objectives of this research were to: (a) test the suitability of the Water Quality Index (WQI) and the Integrated weight Water Quality Index (IwWQI) to assess groundwater quality on a regional scale; (b) assess the spatial distribution pattern (clustered, dispersed, or random type) of groundwater quality using hot-spot analysis; (c) assess the health risk for the population exposed to contaminated water consumption using the Total Hazard Index (THI) and cumulative carcinogenic risk (CCR). The results of this research can also serve as support for development and application of water indices in other areas where groundwater resources are under significant stress.

## 2. Materials and Methods

### 2.1. Study Area

**Figure 1:**
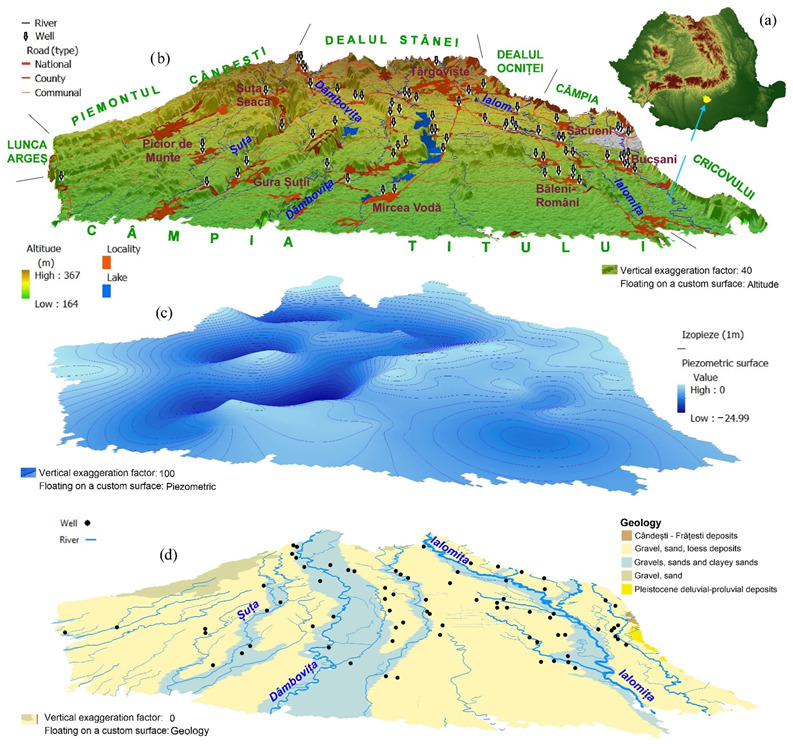
The geographical position of Târgovişte Plain in Romania (**a**), the 3D terrain model and sampled wells (**b**), the 3D model of the shallow groundwater (**c**) and geological map (**d**) (according to Topographic Map of Romania, 1978–1982).

Târgovişte Plain (~1000 km^2^) is located in the southern part of Romania (Figure 1a). The altitude in the area decreases from the north (280 m), at the contact with the Subcarpathian hilly region, towards the south (180 m), at the contact with a subsiding plain. The Târgoviște Plain is of piedmont type, and includes a complex of fluvial terraces (i.e., gravel, sand, and loessial deposits) of the allochthonous rivers Ialomiţa and Dâmboviţa and of the local river network [42].

Dâmboviţa (average annual discharge 10.2 m^3^/s) and Ialomiţa (average annual discharge 7.8 m^3^/s) rivers, the two major watercourses crossing the plain, are arranged approximately parallel in the northern part and divergent in the southern part of the study area. A series of reservoirs has been built (e.g., Văcăreşti on Dâmbovița and Udreşti, Bunget 1 and 2, Brăteşti, Adunaţi, Ilfoveni) on the Ilfov River, a tributary of the Ialomiţa River, to compensate for the extreme intrannual and interannual variability in stream discharges for the river systems in the area.

The boundaries of the surface water catchments generally correspond to the boundaries of the groundwater bodies associated with each catchment, and the structure and lithology of the deposits are relatively uniform. In the Târgovişte Plain hydrographic space, there are two bodies of groundwater (ROAG02; ROIL12). These aquifers are classified as porous and are both set in Pleistocene deposits, with the water table located on average at depths of 2–10 m (ROAG02) and 10–25 m (ROIL12), respectively.

The general climatic conditions (i.e., moderately continental temperate climate, with an average annual rainfall of 600 mm and average annual temperatures of 10 °C) favor the development of luvisols (luvisols and planosols) and alluvial soils. Pseudogleyed planosols and pseudogleyed soils are present, especially in the west of the Dâmbovița Valley, but also in some central areas, on the interfluve between Dâmbovița and Ialomița, while typical luvisols dominate the Dâmbovița–Ialomița interfluve and are less prevalent in the western half of the region. Longitudinal strips of gleyed eutricambosols are present along the main terraces of the rivers, while typical or gleyed alluvial soils are present in the low-lying areas [43].

The region is currently experiencing significant anthropogenic pressure (average density of 200 inhabitants/km^2^). Most of the rural settlements (i.e., 94 settlements present in the Târgovişte Plain) are located along the valleys of the major watercourses (i.e., Dâmboviţa and Ialomiţa) and their tributaries, because these valleys provide fertile soils and easy access to groundwater resources through shallow wells [44]. Land use in the area is dominated by agriculture (64% of the total area). Arable land is mostly used for production of cereals (82% of the agricultural land), with the remainder of the agricultural land being used mostly for pastures and hayfields (14%) and (greenhouse) vegetables [45].

### 2.2. Sampling and Analytical Procedures

All chemical reagents were of analytical grade and in the case of ion chromatography, Fisher Scientific reagents and standards, ACS grade were used. For heavy metal analysis, HNO_3_ 69% was used for acidification and digestion procedures to reach a pH < 2 and thus avoid the presence of precipitating base salts in the sample, which can reduce the metal concentration. In the other analyses, aqua regia (i.e., hydrochloric and nitric acids, high purity, Merck, Kenilworth, NJ, USA) were used for the digestion process. Deionized water, supplied by a MilliQ water purification system (Millipore, Billerica, MA, USA), was used throughout (resistivity of 18 MΩ·cm).

The water samples were collected during the summer from 80 open public wells with water depth between 1 and 25 m (Figure 1b). The sampling was performed according to [46]. The selection of the sampling locations was aimed at achieving a uniform distribution of the sampling locations for the study area. In rural communities with a large number of public wells, 3–5 uniformly distributed sampling locations were selected.

The sampling bottles were rinsed 2–3 times with the target groundwater before samples were collected. After collection, the groundwater samples were prelabeled, refrigerated, and transported at 4 °C to be analyzed for hydrochemical indicators. The samples were filtered through 0.45 μm cellulose membranes and then transferred into prewashed high-density polyethylene (HDPE) bottles. Temperature, pH, electrical conductivity (EC), TDS, and salinity were measured in situ using a Consort 3030 multiparameter.

The acidified samples (pH < 2) were combined in a digestion vessel with the aqua regia (HNO_3_ 67% and HCl 37%). After 10 min of stirring, the samples were digested on a hot plate using a TOPwave microwave-assisted pressure digester (Analytic Jena, Jena, Germany). The clear solutions were transferred with distilled water to volumetric flasks (25 mL). The content of metal, including Cr, Pb, Ni, Zn, Al, Cu, Fe, and Mn, was determined by inductively coupled plasma–mass spectrometry (ICP-MS) using an iCAP™Q ICP-MS spectrometer (Thermo Fisher Scientific Inc., Waltham, MA, USA). The measurements were performed in triplicate in the standard mode (STD), using the Qtegra Intelligent Scientific Data Solution. The relative standard deviation (RSD) values were in the range of 0.01–2.66%. The limits of detection (LODs) and limits of quantitation (LOQs) of analyzed elements were established using the calibration data. Metal calibration curves highlighted a good linearity over the concentration range (0.01 to 10.0 mg/L), with correlation coefficients (r) in the range of 0.991 to 0.999. Accuracy and precision in the ranges of 92–105% and 1–8%, respectively, were considered good in terms of method performance characteristics.

Sulfate (SO_4_^2−^) and bicarbonate (HCO_3_^−^) ion concentrations were measured by titration with a stoppered burette (500 mL). Nitrate (NO_3_^−^) concentrations were assessed by spectrophotometry using an Evolution™ 260 Bio UV-visible spectrophotometer (Thermo Fisher Scientific Inc., Waltham, MA, USA), equipped with a 10 mm path length quartz cell. UV spectra were recorded in the range 190–300 nm. In addition, data were recorded in digitized form with a resolution of 1 nm and the scan rate was 60 nm/min.

Chloride (Cl^−^) concentrations were measured via ion chromatography using a Dionex ICS-6000 HPIC ion chromatography system (Thermo Fisher Scientific Inc., Waltham, MA, USA) equipped with automated eluent generation, self-regenerating suppression and Ion-Pac columns (2 mm), and flow rates 0.2 mL/min for simultaneous analysis of anions (Cl^−^) and cations (Ca^2+^, Mg^2+^, Na^+^, K^+^).

### 2.3. Water Quality Index (WQI)

The Water Quality Index (WQI) proposed by Brown et al. (1970) [25] and subsequently modified by several authors [47,48,49,50,51,52,53,54] was used for determining the sustainability of groundwater resources for drinking and domestic consumption. For calculating and representing the spatial distribution of the WQI, two scenarios were used. Scenario 1 included all the parameters measured either in the field or in the lab (i.e., 19 parameters)., while Scenario 2 covered only the 13 parameters that had high values or were considered to pose the highest risk to the population (NO_3_^−^, Fe, Mn, Cr, Pb, Ni, Zn, Al, Cu, Cd). Ca^2+^, Mg^2+^, Na^+^, K^+^, Cl^−^, and SO_4_^2−^ were excluded from the WQI calculations as they did not exceed the WHO limits or showed good pH and HCO_3_^−^ (Table 1). In both scenarios, the value of the weight (W_i_) was the same.

Thus, WQI was defined as the linear sum of subindices of the various components and was computed for all samples using Equation (1):

(1) $$\mathrm{WQI}=\sum_{i=1}^{n}W_{i}{*q}_{i}$$

where W_i_ is the relative weight (Equation (2)) and q_i_ is the quality rating scale of each parameter.

Relative weight (W_i_) of each parameter is the ratio between the weight of each parameter and the sum of the relative weights of all the parameters considered in the index (Equation (2)).

(2) $$W_{i}=\frac{w_{i}}{\sum_{i=1}^{n}w_{i}}$$

The weight associated with each parameter ranged between 2 and 5 (Table 1) and took into account the degree of risk to which consumers were subjected [55]. Thus, the major anions and cations, which present a low risk for the population, have the lowest weights, i.e., 2–3 (Ca^2+^, Mg^2+^, Na^+^, K^+^, Cl^−^ and HCO_3_^−^), a weight of 4 was assigned to pH, EC, TDS, Fe, Mn) and a weight of 5 for NO_3_^−^ and heavy metals (Cr, Pb, Ni, Zn, Al, Cu), which are considered to represent the highest risk if the values exceed the maximum acceptable concentration limits. For the purpose of this research, the guidelines provided by the WHO (2011) [56] for concentration of contaminants in drinking water were used.

The quality rating scale (q_i_) was determined as the ratio between the value of each parameter measured in the respective sample [mg/L] and the drinking limit according to the WHO standard (2011) (with the exception of pH, for which it was set as 7) (Table 2) [56] (Equation (3)), and is expressed in percentages:

(3) $$q_{i}=\left(\frac{C_{i}}{S_{i}}\right)\times 100$$

### 2.4. Integrated Weight Water Quality Index (IwWQI)

The entropy theory proposed by Shannon (1948) [57] was used to determine the “weight” for each of the 19 parameters used for the calculation of the Integrated weight Water Quality Index (IwWQI) (Equation (4)). For the purpose of this study, IwWQI was considered to be Scenario 3 and was calculated using the following equation (Equation (4)):

(4) $$\mathrm{IwWQI}=\sum_{j=1}^{m}W_{j}Q_{j}$$

where W_j_ is the integrated weight of each parameter and Q_j_ is the quality rating scale.

For the calculation of IwWQI, all values were normalized using the procedure described by Taheriyoun et al., 2010 [58] using the matrix from Equation (5):

(5) $$X=\left[\begin{array}{ccc} x_{11} & \cdots & x_{1n}\\ \vdots & \ddots & \vdots \\ x_{m1} & \cdots & x_{\mathrm{mn}} \end{array}\right]$$

where m represents the total number of well samples (m = 1, 2, …, 80) and n is the number of physical and chemical parameters available for each well (n = 1, 2, …, 19).

The normalized value “y_ij_” (Equation (6)) depends on the maximum and minimum criteria using efficiency type [59] (Equation (6)) and the standard matrix is Y = (y_ij_)(m × n) (Equation (7)).

(6) $$y_{\mathrm{ij}}=\frac{x_{\mathrm{ij}}-{\left(x_{\mathrm{ij}}\right)}_{\min}}{{\left(x_{\mathrm{ij}}\right)}_{\max}-{\left(x_{\mathrm{ij}}\right)}_{\min}}$$

(7) $$Y=\left[\begin{array}{ccc} y_{11} & \cdots & y_{1n}\\ \vdots & \ddots & \vdots \\ y_{m1} & \cdots & y_{\mathrm{mn}} \end{array}\right]$$

The integrated-weight (W_j_) was computed using Equation (8):

(8) $$W_{j}=p\times w_{j1}+\left(1-p\right)w_{j2}$$

where w_j1_ is entropy weight and p is the preference coefficient and p ∈ [0,1]. The terms in Equation (8) are calculated using the following Equations (9)–(13).

(9) $$p=\sum_{j=1}^{n}[{\left(w_{j}-w_{j1}\right)}^{2}+{\left(w_{j}-w_{j2}\right)}^{2}]$$

(10) $$w_{j}=\frac{w_{j1}\times w_{j2}}{\sum_{j=1}^{n}w_{j1}\times w_{j2}}$$

Information entropy (e_j_) and entropy weight (w_j1_) were obtained using Equations (11)–(13):

(11) $$e_{j}=-\frac{1}{\mathrm{lnm}}\sum_{i=1}^{m}y_{i}{\mathrm{lny}}_{j}$$

(12) $$y_{j}=\frac{y_{\mathrm{ij}}+10^{-4}}{\sum_{i=1}^{m}\left(y_{\mathrm{ij}}+10^{-4}\right)}$$

(10^−4^ is used to meaningful the equation; [22])

(13) $$w_{j1}=\frac{1-e_{j}}{\sum_{j=1}^{n}\left(1-e_{j}\right)}$$

(14) $$w_{j2}=\frac{S_{j}}{\sum_{j=1}^{m}S_{j}}$$

(15) $$s_{j}=\delta_{j}\sum_{j=1}^{m}\left(1-r_{\mathrm{ij}}\right)$$

The correlation coefficient (r_ij_) was computed using Equation (16):

(16) $$r_{\mathrm{ij}}=\frac{\sum \left(x_{\mathrm{ij}}-\bar{x_{\mathrm{ij}}}\right)\left(y_{\mathrm{ij}}-\bar{y_{\mathrm{ij}}}\right)}{\sqrt{\sum {\left(x_{\mathrm{ij}}-\bar{x_{\mathrm{ij}}}\right)}^{2}{\left(y_{\mathrm{ij}}-\bar{y_{\mathrm{ij}}}\right)}^{2}}}\;x_{\mathrm{ij}}\;\mathrm{and}\;y_{\mathrm{ij}}\;\mathrm{are}\;\mathrm{the}\;\mathrm{mean}\;\mathrm{values}$$

$\bar{x_{\mathrm{ij}}}$ and $\bar{y_{\mathrm{ij}}}$ are the average values of x_ij_ and y_ij_,

The quality rating scale (Equations (17) and (18)) was calculated according to the concentration of each parameter (C_j_) and expressed in mg/L, the ideal concentration (C_jp_) and the standard value (S_j_) for each parameter according to the WHO [mg/L] [60]. C_jp_ was set as zero (ideal concentration = no contaminants in water), with the exception of pH, for which C_jp_ was set as 7.

(17) $$Q_{j}=\frac{C_{j}-C_{\mathrm{jp}}}{S_{j}-C_{\mathrm{pj}}}\times 100\%$$

(18) $$Q_{\mathrm{pH}}=\frac{C_{\mathrm{pH}}-7}{8.5-7}\times 100$$

### 2.5. Data Interpolation and Hot-Spot Analysis

A deterministic interpolation method (i.e., IDW—Inverse Distance Weighting algorithm) was used to obtain the maps of spatial distribution for each of the hydrochemical parameters [61]. The results of the interpolation were validated using data collected in the field and the associated errors were calculated using the mean error (ME) and the root mean square error (RMSE) [62].

Depending on the values obtained for WQI and IwWQI, the groundwater quality in the study area, for each of the three scenarios, was rated as excellent (WQI < 50), good (50 < WQI < 100), poor (100 < WQI < 200), very poor (200 < WQI < 300), or extremely poor (WQI > 300) [20,63].

Hot-Spot Analysis (HSA) employing Getis-OrdGi* was used to provide additional insights into groundwater quality spatial patterns using *p*-values and *z*-scores for all three scenarios (two for WQIs and one for IwWQI). These indicators offer information regarding the spatial clustering of the features with either low or high values [64]. Spatial autocorrelation was computed using the Global Moran’s I coefficient and allowed for the assessment of the spatial distribution pattern (clustered, dispersed, or random type) of the water quality parameters. A positive value of the Moran’s I index, when the *z*-score or *p*-value has statistical significance, shows clustering, while a negative value relates to dispersion [65].

### 2.6. Assessment of the Impact on Human Health

Assessment of carcinogenic and noncarcinogenic health risks to adults due to ingestion or prolonged contact with contaminants that are present in groundwater can be analyzed, depending on the degree of toxicity, using the total hazard index (THI) and cumulative carcinogenic risk (CCR) for highly carcinogenic metals. For a more accurate assessment, we used specific algorithms to quantify the risks of ingestion or contact with contaminated water using chronic daily intake (CDI) for oral and dermal contact (Equations (19) and (20)). The contact with water was also considered because in the areas with intensive vegetable growing, groundwater is used for irrigation and washing of the vegetable products.

(19) $${\mathrm{CDI}}_{i}\mathrm{oral}=\frac{C_{i}*\mathrm{IR}*\mathrm{EF}*\mathrm{ED}}{\mathrm{BW}*\mathrm{AT}}$$

(20) $${\mathrm{CDI}}_{i}\mathrm{dermal}=\frac{C_{i}{*\mathrm{SA}*K}_{p}*\mathrm{ET}*\mathrm{EF}*\mathrm{ED}*10^{-3}}{\mathrm{BW}*\mathrm{AT}}$$

where C_i_ is the concentration of ith toxic materials, IR is the ingestion of rate of drinking water (2 L for adults), EF is the exposure frequency (365 days/year for oral and 350 days/year for dermal), ED is the exposure duration (70 years for oral and 30 years for dermal), SA is the surface skin (18,000 cm^2^), ET is the exposure time—oral (0.58 h/day), K_p_ is the dermal permeability coefficient (cm/h) (Table 3), BW is the average body weight (70 kg), and AT is the average time (25,550 days for oral and 10,950 days for dermal).

It is possible to evaluate the hazard quotient (HQ) for each constituent in part using the ratio between the CDI_i_ and reference dose (RfD_i_) (Table 3) (Equation (21)) or to cumulate the values using THI, including both oral and dermal exposure (Equation (22)).

(21) $${\mathrm{HQ}}_{i}=\frac{{\mathrm{CDI}}_{i}}{{\mathrm{RfD}}_{i}}$$

(22) $$\mathrm{THI}=\sum_{i=1}^{n}({\mathrm{HQ}}_{i\;\mathrm{oral}}+{\mathrm{HQ}}_{i\;\mathrm{dermal}})$$

Value THI > 4 of cumulative noncarcinogenic multiple contaminants from water indicate a high health risk, 4 > THI > 1 indicate a medium health risk, 0.1 < THI < 1 a low health risk, and for THI < 0.1 there is no health risk for the population [66].

Prolonged consumption of water with a high content of heavy metals has a high carcinogenic potential that can be assessed using CR_i_ (carcinogenic risk) (Equation (23)) for each metal (Equation (5)). The value of the cancer slope factor (CSF) for Pb, Cr, and Ni as heavy metals used in our study are 0.5 and 1.7, respectively 0.0085 (mg kg^−1^ day^−1^).

(23) $${\mathrm{CR}}_{i}={\mathrm{CDI}}_{i}\times {\mathrm{CSF}}_{i}$$

Cumulative carcinogenic risk (CCR) was calculated using Equation (24) for 35-year duration of exposure, where n is the number of assessed carcinogens considered (Pb, Cr, Ni). CCR values > 1.0 × 10^−4^ indicate a high possibility of developing cancer, 1.0 × 10^−6^ < CCR < 1.0 × 10^−4^ indicate an acceptable risk, and CCR < 1.0 × 10^−6^ indicate no carcinogenic threats to health [67].

(24) $$\mathrm{CCR}=\sum_{i=1}^{n}{\mathrm{CR}}_{i}$$

## 3. Results

### 3.1. Hydrogeochemistry and Trace Element (TE) Evaluation

The chemical composition of groundwater in the Târgovişte Plain is controlled by the complex interaction of environmental factors and anthropogenic activities. Previous findings indicate that the interactions between these factors vary in space and time [21,68].

**Figure 2:**
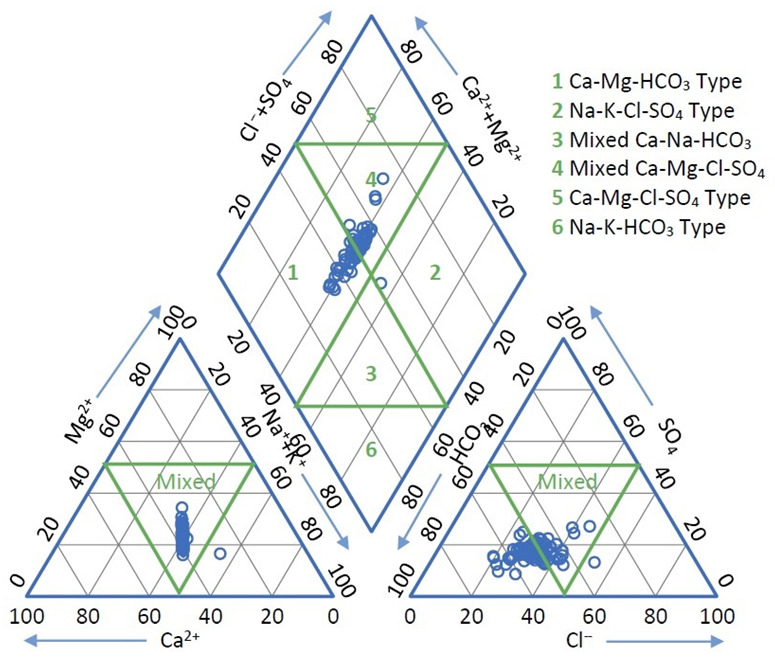
Piper diagram for groundwater samples collected from Târgovişte Plain.

A trilinear Piper diagram [69] was used to interpret the hydrochemical characteristics of groundwater (Figure 2) and to explain the variation in concentrations of anions and cations. Two main classes of hydrochemical type can be discerned: a Ca^2+^–Mg^2+^–HCO_3_^−^ type and a mixed type Ca^2+^−Mg^2+^−Cl^−^−SO_4_^2^, with only one of the samples belonging to a Na^+^–K^+^–Cl^−^–SO_4_^2^^−^ type. There is a slight dominance of HCO_3_^−^ anions followed by Cl^−^, NO^3^^−^ and SO_4_^2^^−^, while the results are mixed for the cations, with Ca^2+^ closely followed by Na^2+^, Mg^+^ and K^+^ (Figure 2).

**Figure 3:**
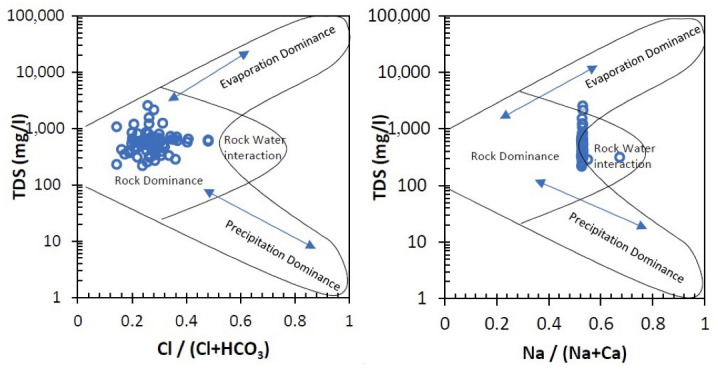
Gibbs diagram for groundwater samples collected from Târgovişte Plain.

To identify the sources of the main chemical constituents in the shallow groundwater, a Gibbs diagram [70], showing the ratios between TDS and Cl^−^/(Cl^−^+ HCO_3_^−^) and TDS and Na^+^/(Na^+^+Ca^2+^), respectively, was used (Figure 3). The Gibbs diagram is used to link the water composition to the aquifer lithological characteristics by indicating if the rock, rock–water interaction, evaporation, or precipitation is dominant in controlling the water composition. For the Târgovişte Plain, the Gibbs diagram shows that the composition of almost all the samples fell under rock dominance or at the limit between rock and rock–water dominance area, with several of the samples positioned in the evaporation dominance area of the diagram. Ca^2+^/Mg^2+^ ratios greater than 2 indicate that the presence of Ca^2+^ and Mg^2+^ ions in all samples (only 3 samples had values less than 2) is the determinant of hydrolysis of silicate minerals. The Cl^−^/Na^+^ ratios are generally below 1, which indicates silicate minerals as a source of Na^+^ release into the groundwater. Very strong positive correlations between Ca^2+^, Mg^2+^, and Na^+^ (0.92 < R^2^ < 0.98) (Table 2) indicate that the respective ions are involved in numerous chemical reactions related to the oxidation-reduction process and ion exchange [71]. The Ca^2+^, Mg^2+^ and HCO_3_^−^ in the analyzed area have similar geographic distribution, suggesting that the sources of water for the aquifer are the same or similar [8], while the much more varied distribution of Cl^−^ and SO_4_^2^^−^ suggests a high influence of local conditions, such as land use and other anthropogenic activities. In the V-NW part of the study area, at the contact with the Subcarpathian hills, the hydrochemical characteristics of the groundwater are influenced by water coming from slope runoff or from the springs at the base of the slope. The results indicate that oxidation processes of sulfides in rocks led to an increase in the amount of iron released into groundwater, while the reduction processes of sulfates that take place in the presence of organic matter led to an increase in S values and the formation of HCO_3_^−^.

A chloro-alkaline index (CAI) was also used to assess the degree of exchange between the base ions [72,73]. CAI values are calculated using the following formulae:

(25) $$\mathrm{CAI}\;1=\frac{\mathrm{Cl}-\left(\mathrm{Na}+K\right)}{\mathrm{Cl}}$$

when the ratio is positive, it indicates the exchange of Na^+^ and K^+^ from the water with Mg and Ca from the rock, and for

(26) $$\mathrm{CAI}\;2=\frac{\mathrm{Cl}-\left(\mathrm{Na}+K\right)}{{\mathrm{HCO}}_{3}+{\mathrm{SO}}_{4}+{\mathrm{CO}}_{3}+{\mathrm{NO}}_{3}}$$

when the exchange is reversed, the ratio is negative (the ratios are expressed in meq/L).

The values of CAI 1 and CAI 2 correlate very well (r = 0.95) and range between −1.84 and 0.45, which indicates reduced exchanges between the Na^+^ and K^+^ in the water with Mg^2+^ and Ca^2+^ in the rock in 36.25% of cases (29 samples). In 63.75% of the samples, the calculations indicate a negative ratio (51 samples), indicating the reverse processes. However, the positive values found in areas with intensive vegetable cropping (Băleni, Comişani settlements) can also be caused by fertilizers and other agricultural chemicals, which are intensive in the respective areas.

Regarding pH, the samples showed neutral values between 6.5 and 7.3, without exceeding the WHO guidelines. TDS values are 217–2550 mg/L. Values <600 mg/L in water are considered acceptable for consumption, while water with TDS values > 1000 mg/L is unpalatable due to the accentuated taste and sometimes unpleasant odors. Only 7 samples showed TDS values over 1000 mg/L, while 27 samples were 600–1000 mg/L. The remaining samples (57%) had TDS values below 600 mg/L.

Nitrate (NO_3_) is currently one of the most common groundwater pollutants, especially in shallow groundwater due to both natural and anthropogenic processes. Generally, nitrate concentrations in groundwater vary little over time, being less sensitive compared to nitrate in rivers. The increase in nitrate concentration is due to microbial nitrification processes or synthetic fertilizers used in excess, animal manure, or lack of sewerage systems. Of the analyzed samples, 15% showed high values of NO_3_ (>50 mg/L), especially on the surfaces used in intensive vegetable growing (Băleni, Lazuri, Comişani), with small depths of groundwater (1–4 m) (Bucşani, Bungetu, Brăteştii de Jos) or near the accumulation lake from Pierşinari (Văcăreşti, Lucieni, Pierşinari). Although the consumption of nitrate water (NO_3_) alone is not carcinogenic, endogenous nitrosation is probably carcinogenic to humans (Group 2A). At the same time, consuming water with high levels of nitrates is an important risk factor for methemoglobinemia, especially for infants. If the source of contamination comes from animal manure or septic tanks/sewerage networks, nitrate ingestion is most often associated with microbial contamination, which will cause gastrointestinal infections.

The average concentrations of trace elements in groundwater from Târgovişte Plain are in the order Al > Zn > Ni > Pb > Cu > Fe > Mn > Cr. All are moderately abundant (0.1–0.001 mg/L) and, on average, Ni, Fe, and Pb are higher than WHO standards (see Table 1). Heavy metals become hazardous when they are accumulated in the body in high concentrations, and in cases of some toxic elements (chromium, nickel, and lead) increase the risk of developing various diseases (cardiovascular diseases, hypertension, adult increase in systolic blood pressure), allergies on dermal contact or various forms of cancer. Of these, Cr and Ni are common elements in Earth’s crust, but are also associated with anthropogenic activities. Pb is rarely found in water from natural sources as an effect of dissolution and the main cause of accumulation is anthropogenic activity.

The spatial distributions of the 3 TEs are similar, the areas with high values being recorded along the rivers Dâmboviţa (Lucieni, Pierşinari, Văcăreşti) and Ialomiţa (Bucşani, Comişani, Lazuri) as an effect of accumulation in sediments and transfer in groundwater. The other two main sources of contamination are the Târgovişte industrial area (an important steel center in Romania and the related tailings stops) as well as the use of pesticides and herbicides in agricultural activities. Recent studies show that glyphosate-based herbicides (GBH) contain Cr, Ni, Pb, and other heavy metals [74], while different fertilizers (copper sulfate and iron sulfate) contain the highest concentrations of Pb and Ni [75]. In Romania, glyphosate has been banned since 2017, but unfortunately it is still on sale in online stores and beyond.

The average nickel (Ni) level in the Târgovişte Plain is 0.033 mg/L, concentration 0.0009—0.087 mg/L, and 68% of the samples exceed the WHO recommendations and the national drinking standards of 0.02 mg/L.

Lead (Pb) has an average concentration of 0.06 mg/L, higher than the WHO guideline recommendation, the values being between 0.0001 and 0.06 mg/L, with 36 samples having values higher than the allowed limits.

Chromium (Cr) concentration ranged from 0.01 to 0.09 mg/L with a mean of 0.034 mg/L, and 17 of the samples collected contained exceeded 0.05 mg/L according to the WHO guide.

### 3.2. Water Quality for Human Consumption

An advantage of water quality indices is that they provide an integrated perspective on the water quality and can target specific concerns related to the groundwater body of interest. Hence, indices provide an extremely useful tool for policymakers or water managers to assist the development of policies and/or strategies aimed at the protection of groundwater bodies.

The WQI (Equation (1)), was used to assess the quality of groundwater used by the population for drinking and domestic activities. The use of the WQI/IwWQI indices simplifies the understanding of the values of all the physicochemical parameters analyzed by transforming them into a single value [76].

The two indices (WQI and IwWQI) were selected for comparative analysis because they both use similar calculation methods, including being based on the summation of subindices and share the same water quality classes (WQI < 50 good; 50 < WQI < 100 poor; 100 < WQI < 200 very poor; 200 < WQI < 300 extremely poor; WQI > 300).

The number of parameters used for Scenario 2 is similar to the number of parameters used in most of the previous studies, which range between 8 and 11 [38]. In our research, the number of parameters used was higher due to the addition of heavy metals, which are not typically included in similar water quality indices.

**Figure 4:**
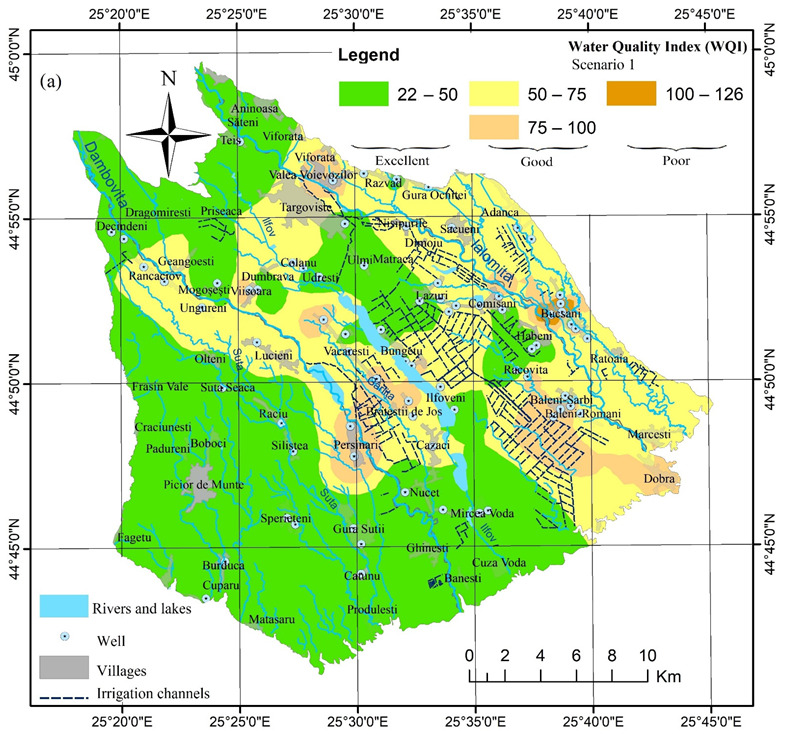

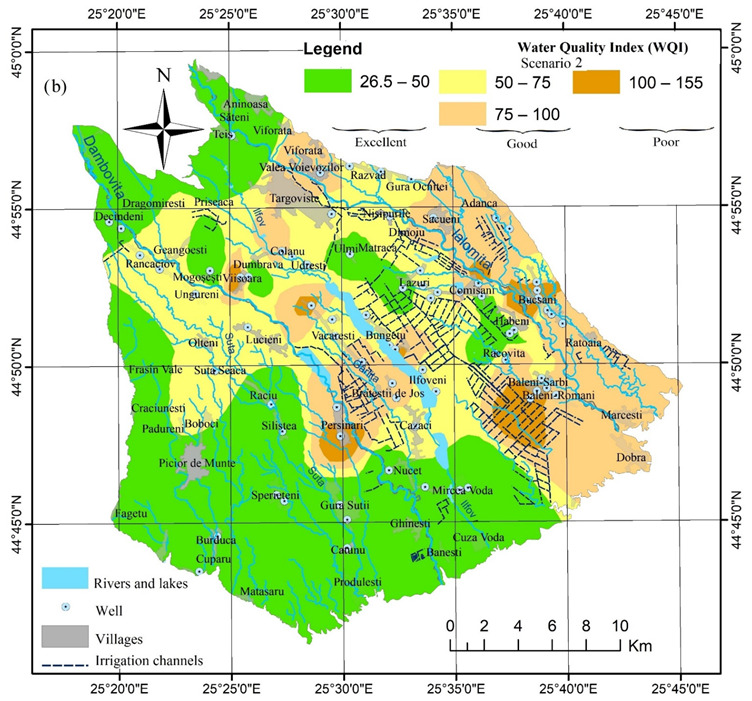
Maps of the Water Quality Index (WQI) in Târgovişte Plain in Scenario 1 (**a**) and Scenario 2 (**b**).

The WQI and IwWQI values show the relationships between the spatial distribution of values, the depth of the groundwater level, land use, and anthropogenic activities (i.e., intensive agriculture and industrial activities). Thus, for WQI in both scenarios (Figure 4) the areas with high groundwater depth (>15–25 m), distributed mainly west of the Dâmboviţa River and in the north of the study area at the contact with the Subcarpathian Hills have excellent groundwater quality (see also Figure 1), while in the central area, south of Târgovişte or in areas with shallow water depths (Bucşani, Lazuri, Comişani) and very intense agricultural activities (Băleni) have poor groundwater quality.

The use of the two scenarios highlights the importance of proper selection of the parameters included in the WQI calculation to avoid the phenomenon of “eclipsing.” Thus, based on Scenario 1, 48.7% of the sampled wells (39 wells) were in the excellent category, 47.5% of the wells (38 wells) were in a good category, and only 4% of the wells (3 wells) were in the poor category. In Scenario 2, which used the reduced number of 13 parameters compared to the 19 used in Scenario 1, the percentage of wells in the poor category increased four times to 16% of wells sampled (13 wells), while 50% of the wells (i.e., 40 wells) were in a good category and 33% of the wells (27 wells) were in the excellent category (Table 4). Overall, Scenario 2 reflected better the quality of groundwater because of the high values of Pb, Ni and NO_3_^−^ in some wells where the maximum values as per the WHO guidelines were exceeded; this led to the classification of these wells as poor. However, in the first scenario, the WQI values in those wells were very close to the good/poor limit (95–99—poor limit 100) but the increase by a few percent of the weight of these elements led to these marginal wells being classified as poor.

**Figure 5:**
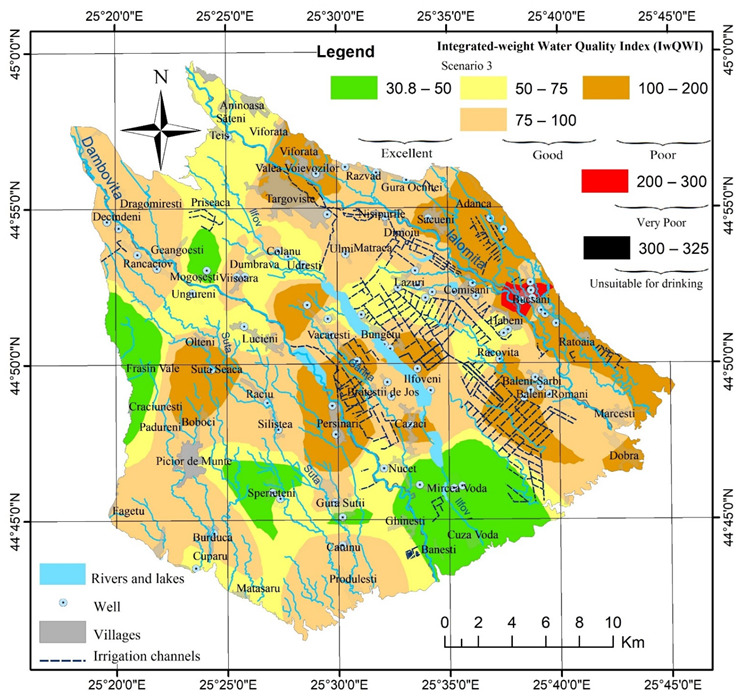
Map of Integrated weight Water Quality Index (IwWQI) in Târgovişte Plain.

For Scenario 3 (i.e., IwWQ), the distribution of the water quality in the sampled wells across the various categories (i.e., Figure 5) was consistent with the results obtained in both Scenarios 1 and 2 for most of the study area. Scenario 3 reflects better the quality of the groundwater in the studied area. Thus, the number of wells with excellent and good water quality decreased by 17 wells (75% decrease compared to Scenario 1 and a 7 well decrease compared to Scenario 2, respectively), while the number of wells with poor water quality or water unsuitable for drinking increased by 25% (20 wells) compared to both Scenario 1 and Scenario 2. The extent and position of the areas with poor quality and very poor quality are similar to those in the previous scenarios; however, Scenario 3 better highlights the relationships between hydrogeological characteristics, groundwater depth, land use, and anthropogenic influences.

Notably, the integrated weight values calculated using the entropy approach (Table 1) resulted in higher index values compared to Scenario 1 and Scenario 2 for EC (0.4053) and TDS (0.2121), indicating that these parameters provided the largest effective information, while the values for HCO3^−^ (0.017), SO_4_^2−^ (0.0089), and pH (0.0047) were smaller, suggesting that they provided the least effective information.

The quality of the shallow groundwater in the Târgovişte Plain is similar to that of other water bodies in Romania [77,78] and is expected to improve over the medium and long term once sewerage networks and treatment plants are put into operation in rural areas. In the meantime, it is difficult to compare the results of our study with previous groundwater quality studies in the area as the latter have a very coarse spatial resolution (i.e., regional values) and treat parameters individually rather than compounding their concentrations into an index. For example, nitrate contamination of groundwater is frequently discussed in other studies [79,80,81,82]; however, the respective studies are lacking the inclusion of heavy metals or vice versa.

In order to highlight the importance of each parameter and its contribution to the total value of WQI/IwWQI, the effective weight (E_wi_) of each parameter was also calculated (Equation (27)). The value of each parameter is expressed as a percentage and was calculated as the ratio between the subindex of the respective parameter (SI_i_) and the total value of WQI/IwWQI:

(27) $$E_{\mathrm{Wi}}=\frac{{\mathrm{SI}}_{i}}{\mathrm{WQI}}\times 100$$

The effective weight parameter values (E_wi_; Table 5) suggest that for Scenario 1 and Scenario 2, two of the parameters (i.e., Pb and Ni) cumulatively provide on average ~34–36% of the WQI value, while only 10–13% of the WQI value is provided by the cumulated contributions of NO_3_^−^, EC, pH, TDS and Fe. The rest of the hydrochemical elements have an insignificant weight for the WQI calculation, with the average concentration values of these parameters being below the WHO potability guideline. In the case of IwWQI, the most important contributions are provided by physicochemical parameters (i.e., EC, pH, TDS) followed by Pb and Ni. The presence of heavy metals is closely related to historical pollution from steel plants and the tailings dumps located near Târgovişte city [83].

The accumulation of nitrogen and heavy metals in the riverbed and/or lake sediments from the riverbed together with exchanges at the groundwater–surface water interface determine the poor quality of the groundwater in the villages Viişoara, Văcăreşti, and Pierşinari. On both sides of the Dâmboviţa River, near the accumulation from Pierşinari, the WQI/IwWQI values are high and indicate poor quality of groundwater. On the other side, even if nitrogen can naturally occur in groundwater, the high values in the Băleni and Comişani areas, seem to be related to the intensive vegetable production in these areas, while in the Bucşani area they could be the result of significant leakage from septic tanks considering the very shallow depth of groundwater (1–3 m).

Sensitivity analysis proposed by [84] and modified by [20] (Equation (28)) has been employed for assessing the stability of the results obtained with the three scenarios. Based on this methodology, a higher sensitivity value (S_i_) indicates a more unstable solution.

(28) $$S_{i}=\frac{\left(\frac{V_{i}}{N}\right)-\left(\frac{v_{i}}{n}\right)}{V_{i}}\times 100\%$$

where V_i_ is the WQI or IwWQI value of i-th evaluated well, v_i_ is the WQI or IwWQI value after removing i-th input index (chemical component), N (N = 19) and n (n = 18) are the number of physicochemical parameters when calculating V_i_ and v_i_.

The individual analysis of each well regarding the positive or negative values of sensitivity (S_i_) shows the effect of each physicochemical parameter on the final quality index (WQI or IwWQI) derived for each sample, and thus how a parameter influences the positioning of that well in the quality class in which the water sample falls.

**Figure 6:**
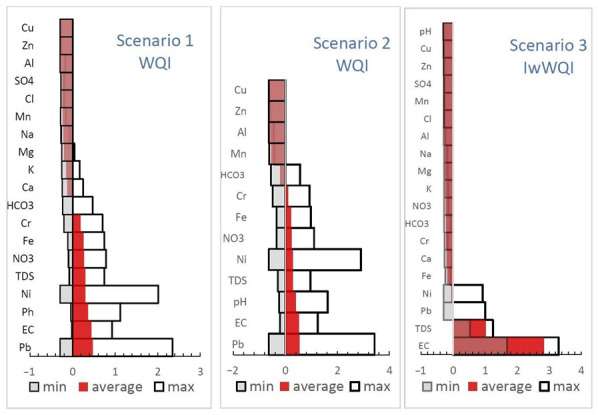
Sensitivity analysis (S_i_) of removing each parameter on the score of WQI (Scenario 1 and 2) and IwWQI (Scenario 3) (values were ordered in descending order).

For Scenarios 1 and 2, the average values (positive/negative) are close and do not exceed ± 0.5%, while for Scenario 3 the positive values of EC and TDS are the main drivers that determine the classification of a well sample in a class of poor quality. However, in the case of average values (Figure 6), it is known that the arithmetic mean is sensitive to extreme values; moreover, the same parameter may have both negative and positive values, in which case the average value becomes insignificant (see the case of Pb and Ni).

**Figure 7:**
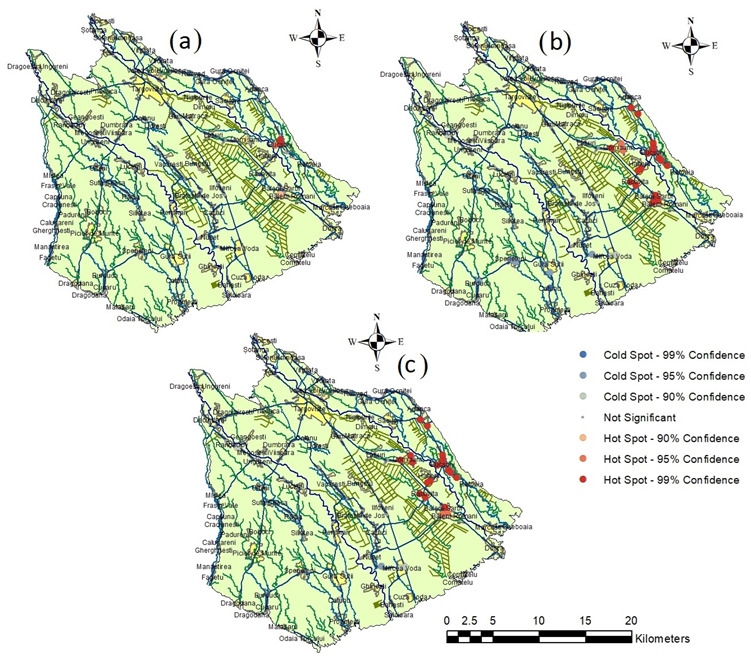
Hot-spot analysis for the eastern part of Târgovişte Plain for each of the three scenarios: (**a**) WQI scenario 1; (**b**) WQI scenario 2; (**c**) IwWQI scenario.

Figure 7 shows the results of the HSA for all three scenarios. All three scenarios provide hot spots (99% and 95% confidence) for analyzed wells in the eastern part of the plain. The analysis showed that most of the wells are cold spots except in the areas with intensive vegetable cropping located in the east. The spatial autocorrelation using Global Moran’s I showed that the overall pattern does not appear to be significantly different from random for all three scenarios.

HSA for WQI Scenario 1 did not find a large area in which wells qualified as hot spots. Only the wells from Bucşani village were classed as hot spots with 95 or 99% confidence, while wells in intensive cropping areas around Băleni were classed as hot spots with 90% confidence. HSA for WQI scenario 2 and for IwWQI (scenario 3) identified more wells as hot spots, with few differences between them. HSA for IwWQI showed 99% hot spots in Comişani and lower-confidence hot spots for the Băleni wells. In contrast, the WQI scenario 2 showed the reverse pattern. Overall, both villages are located in intensive vegetable cropping areas that use irrigation with groundwater on a large scale. Hot-spot analysis shows that IwWQI is a more sensitive indicator that can discriminate better the water quality from neighboring wells compared to the WQI scenarios.

### 3.3. Human Health Risk Assessments

Given that the WQI/IwWQI show that 16–25% of the wells in the Târgovişte Plain fall into the categories poor, very poor, or even unsuitable for drinking, it is necessary to assess the risks to which the population is exposed. These include the cumulative risk of developing cancer due to exposure to carcinogenic metals that are present in groundwater (Ni, Pb, Cr). Ingestion of heavy metals is also dangerous because it influences neuromotor development, and can cause cardiovascular diseases [85,86,87,88].

In our study based on CDI oral and dermal values, we evaluated HQ for one anion (nitrate) and for metals: Mn, Ni, Fe, Cu, Al, Zn, Cr, and Pb (RfD and SF were established according to USEPA standard doses 2004 [67]). Analyzed individually, the average HQ values for each chemical constituent did not exceed the value of 1. However, HQ oral [?] values > 1 were recorded in 2 samples for NO_3_ and 3 samples for Pb, with the values > 1 being recorded in Băleni and Bucşani villages, communities that also have the worst water quality according to the IwWQI (scenario 3) or WQI (scenario 2). Contact with contaminated water does not present a risk to the population, the HQ dermal values being very low (Table 6).

**Figure 8:**
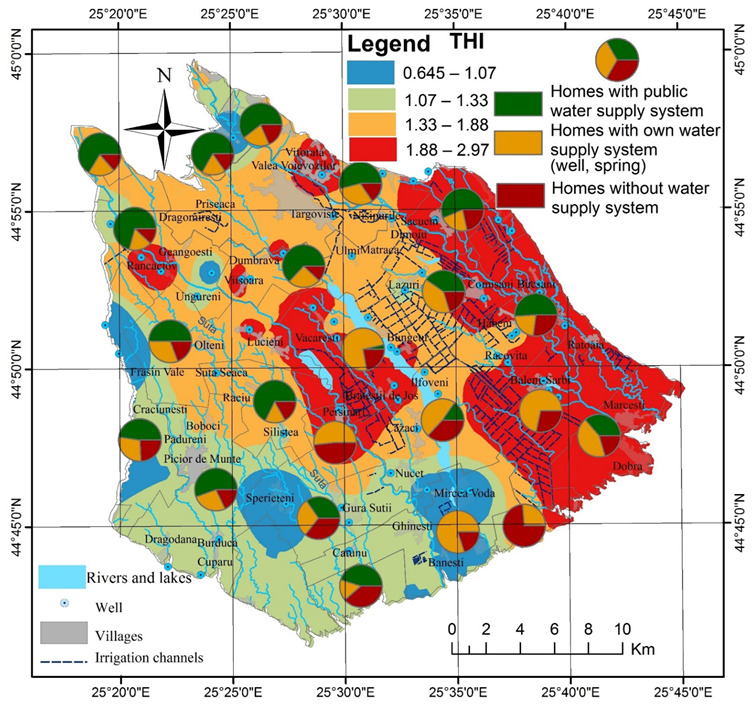
Spatial distribution of health risk (THI) in Târgovişte Plain.

The cumulative values for oral and dermal HQ led to the creation of the Total Hazard Index (THI) map (Figure 8). THI values of cumulative noncarcinogenic risk of multiple contaminants indicated that 80% of the samples have an average health risk (1 < THI < 2.97) for the population that consumes water from underground sources for a long period of time, the most affected being Bucşani, Băleni, Comişani, Pierşinari, and Văcăreşti. The average value of the THI is 1.47 and the areas, with values higher than 1 indicating moderate exposure of approximately 43% of the rural population in the Târgovişte Plain (the city of Târgovişte was not considered in this figure), the areas overlapping those with poor, very poor values or unsuitable from the WQI (scenario 2) and IwWQI (scenario 3).

**Figure 9:**
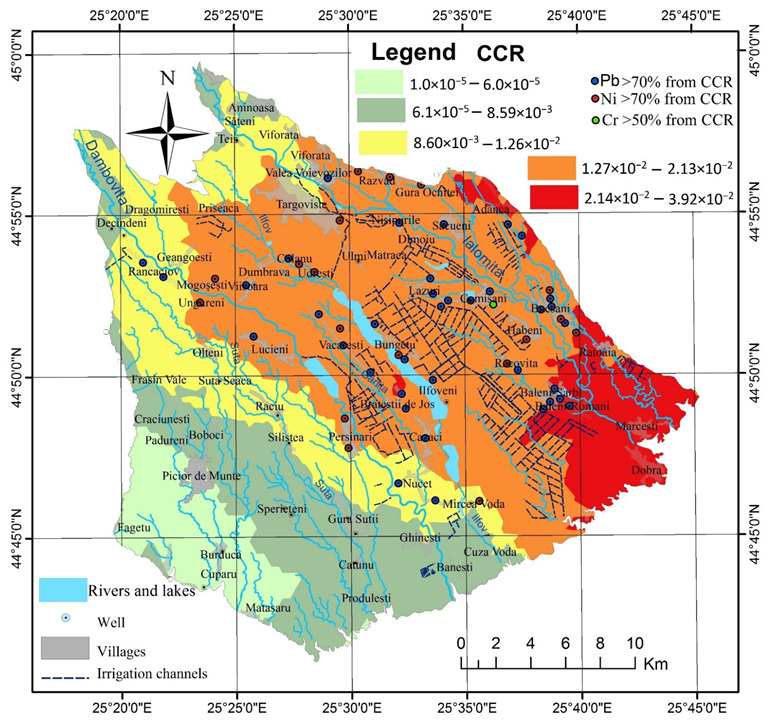
Spatial distribution of cumulative carcinogenic risk (CCR) in Târgovişte Plain.

The cumulative carcinogenic risk (CCR) assessment presented in Table 7 indicates a high value of 1.15 × 10^−2^ compared to values considered “acceptable” 1.0 × 10^−6^ < CCR < 1.0 × 10^−4^, and the order of the mean carcinogenic risk is Ni > Pb > Cr. In 56% of the collected samples, Ni values are dominant, in 43% Pb is the dominant carcinogenic element, and only in 1%, Cr dominates in the calculated value of CCR. Ni represents > 70% of the CCR value in 18 of the samples and Pb concentrations exceed 70% in 13 samples (Figure 9). High CCR is found in the areas where IwWQI suggests poor/very poor groundwater, with Pb being the chief risk mainly in the eastern part, in areas with intensive vegetable growing, while Ni is the chief risk along the Dâmboviţa River and in the south of Târgovişte municipality. This pattern is also mentioned in other studies [40,89] in which high values of carcinogenic elements are found along the main hydrographic channels and increase from upstream to downstream.

The high values, both individually for each carcinogen and in sum, indicate a very high risk of cancer occurrence during life for the population that consumes water from underground sources. The spatial distribution of CCR indicates extremely high values of risk in the villages of Băleni, Bucşani, Brăteştii de Jos, and Bungetu with risk rates of 2 to 4%. Almost 43% of the rural population in the Târgovişte Plain is in areas at risk > 1.12 × 10^−2^, which, compared to the average value of CCR, represents a number of approximately 600 people likely to be diagnosed with cancer during their lifetime if they use groundwater as the main sources for drinking water. The risk of increased cancer incidence and mortality is accentuated by the fact that in 2016, out of the 43,643 homes in the villages of Târgovişte Plain, 41.8% had a water supply connected to a public network, 33.6% had their own water source (fountain, spring), and 24.53% of the houses did not have water supply installations. Under these conditions, 2400 homes do not have access to a verified water source, so approximately 68,000 residents are at risk. The spatial distribution of these dwellings related to the distribution of IwWQI, THI and CCR shows that 12,380 dwellings and approximately 33,426 inhabitants are in villages with poor groundwater quality and a high risk of developing various health conditions associated with consumption of low-quality water. Thus, relating these values to the average value of CCR, it results that 385 people are likely to develop cancer as a result of consuming groundwater contaminated with heavy metals.

The CCR values are directly proportional to the intensive use of agricultural lands and inversely proportional to the depth of the groundwater layer, the distance from Târgovişte municipality, and from the main hydrographic network. Also, the transfer of water from the Pierşinari dam, on the Dâmboviţa River, through derivations in the lakes on the Ilfov valley, determines increases in CCR in the villages located on their river banks (Brăteştii de Jos, Ilfoveni, Bungetu).

**Figure 10:**
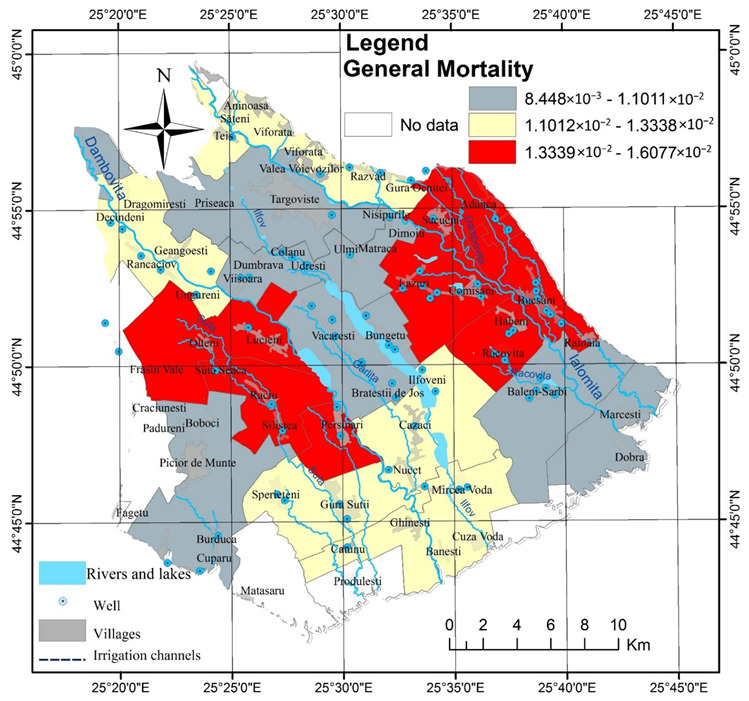
General mortality isn 2019 by administrative units in Târgovişte Plain.

It is difficult to determine the impact of the quality of groundwater consumed by the inhabitants of the rural area of Târgovişte Plain, because there is no database that allows us to evaluate the number of diseases by administrative units; however, the number of real cases of the most common diseases and the mortality rates do correlate with the theoretical values obtained by this analysis. For example, the assessment of general morbidity expressed by the incidence and prevalence of cases both at a national level and in Dâmboviţa County gives us general indication of the health of the population (Figure 10) and the distribution of villages with the highest mortality in the eastern study area and along the Dâmboviţa River overlaps with the areas with poor water quality, THI > 1 and high CCR.

According to a national report on the health of the population (2020) [90] the number of cases of malignant tumors in the records of oncology offices increased in Romania from 430,846 (in 2011) to 510,819 (in 2020), with prevalence increasing from 2.14 × 10^−2^ inhabitants in 2011 to 2.65 × 10^−2^ in 2020. In Romania, in 2020, 161,925 patients with malignant tumors were discharged, which represents a hospitalized morbidity rate of 8.40 × 10^−3^ (4.70 × 10^−3^ in Dâmboviţa county) but the value must be analyzed in the context of COVID−19, because in 2019 the number of cases was 1.28 × 10^−2^ (6.882 × 10^−3^ in Dâmboviţa county). The incidence rate of digestive tract diseases in Romania in 2020 was 8.94 × 10^−2^ inhabitants, and the mortality rate of patients with this condition in Dâmboviţa was 7.34×10^−4^ inhabitants, higher than the national average of 6.92 × 10^−4^ inhabitants.

The values recorded in Romania and in Dâmboviţa County, which were higher than the average value of computed CCR, suggest that some of these cases of tumors or diseases of the digestive tract may be related to ingestion, over a long time, of heavy metals in water but new studies will be needed to confirm this hypothesis.

## 4. Discussion

The number of studies regarding water quality indices is large; most of them evaluate spatially, temporally or spatiotemporally the quality of water using one index while studies that compare the same set of physicochemical parameters (including heavy metals) using different indices are limited.

The use of water quality indices represents a solution when comprehensive datasets are available; however, there are limitations regarding their “universal” application when the differences in the spatial distribution of contaminants, the changes in the set of parameters included in national monitoring programs, and also the scientific advancements relative to the assessment of the inherent risks of consuming contaminated water are considered.

In the case of our study, including all the parameters determined (including heavy metals) in the WQI calculation, resulted in an eclipse of the values that exceeded the drinking limits and the inclusion of more samples in the good-quality class. This problem is well known in the literature [36,38,91,92,93], but is still found in a few studies [35,94].

The applicability of groundwater quality using WQI/IwWQI presented here is somewhat limited due to the exclusion of coliforms (E. coli, total coliform) and pesticides in the calculation of the final values. To account for the fact that shallow groundwater from rural areas is highly exposed to pollution from a series of domestic sources including septic tank leakage and leakage from sewerage transport networks, future quality indicators should include other categories of substances, such as pharmaceuticals, personal care substances, and artificial sweeteners due to their potentially adverse effects on human health [95,96,97,98,99,100], even if this would lead to an increase in the cost of the monitoring program. Thus, for example, in the area of Bucşani, the presence of pit latrines for each house, the lack of a sewerage network and the shallow water table depth (1–3 m below ground) could explain the high nitrate values; however, the inclusion of artificial sweeteners and coliforms would provide clear evidence to support this interpretation.

Without being able to give a decisive answer to the question “What is the best index?” we can say with certainty from the WQI/IwWQI comparison that the use of entropy in the calculation of “integrated weight” and IwWQI led to better results, avoided the eclipse of high values and led to a clear highlighting of areas with poor and very poor quality, unsuitable for drinking in Târgovişte Plain. HSA results support these findings, showing that IwWQI is a more sensitive indicator that can discriminate better the water quality from neighboring wells compared to WQI scenarios.

Although we cannot say with certainty that the high values of morbidity/mortality from the rural area of Dâmboviţa County (with the Târgovişte Plain as a component part) are caused by the poor quality of the consumed water, requiring additional studies to show the links between heavy metals ingested, water and the causes of cancers or digestive disorders, we cannot fail to notice the close values of the calculated CCR and the number of patients with cancer or digestive disorders registered in the medical offices or hospitalized in the region.

Differentiated analysis by age-groups (adults/children) and sex (men/women) regarding CCR and THI, and the extension of the number of carcinogenic contaminants that will be analyzed and included in their calculation are two areas where further research is recommended. Also, the evaluation of the impact of drinking water quality on the health status of the population correlated with WQI/IwWQI must be analyzed bidirectionally in cases of extreme values (very high/very low) of physicochemical parameters included in the calculation, because excellent quality determined by very small values is not necessarily good for the health of the population (for example 20–40% of the daily requirement of Mg comes from water and its deficiency increases the risk of morbidity of newborns). Finally, a transdisciplinary approach to groundwater quality issues that incorporates public health experts along with hydrogeologists and chemists is also recommended.

## 5. Conclusions

In Târgovişte Plain, the quality of shallow groundwater and the risk of developing cancer and other diseases due to water consumption is directly proportional to the intensity of agricultural land use and inversely proportional to the depth of the groundwater layer, the distance from the main hydrographic network/reservoirs, and the distance from the main city (Târgovişte).

The Water Quality Index (WQI) and Integrated Weight Water Quality Index (IwWQI) were used to evaluate groundwater quality. The WQI is often used in the literature because it has the advantage of an easy-to-apply formula, but in the absence of careful selection of parameters or limitation of the number of parameters used, the results of this index become insignificant. The high values of some physicochemical parameters are “hidden” and the high values of these compounds are “eclipsed”, which can cause the sample to be placed in a “good” or “excellent” category even if the individual values far exceed the permissible drinking limits. The IwWQI is a more complex method of calculating water quality, but has the advantage that it reduces the possibility of eclipsing values.

HSA of the WQI in Scenario 1 did not find a large area in which the wells qualify as hot spots, but HSA of the WQI (Scenario 2) and the IwWQI (Scenario 3) provided better results by identifying more wells as hot spots with few differences between them. Hot-spot analysis shows that the IwWQI is a more sensitive indicator that can discriminate better the water quality from neighboring wells compared to WQI scenarios.

In Târgovişte Plain, areas with poor and very poor groundwater quality according to the WQI/IwWQI overlap with moderate risk to human health (THI > 1) for noncarcinogenic contaminants, and the population that does not have access to a verified water source has a 1.15% risk of developing cancer according to the CCR average value (1.1545 × 10^−2^).

The complex analysis of groundwater quality using quality indices, health-risk indicators for the population, validated by hot-spot analysis, and compared to the potential morbidity/mortality rates is an approach with practical applicability that can allow public authorities, policymakers, and health services to develop an integrated approach, leading to the implementation of an efficient monitoring program and optimization of anthropogenic activities in order to prevent groundwater contamination and finally improve the quality of life of the population.

## Figures and Tables

**Table 1:** Coefficients of determination for groundwater parameters.

	pH	EC [μS/cm]	TDS [mg/L]	SO_4_^2−^ [mg/L]	Cl^−^ [mg/L]	HCO_3_^−^ [mg/L]	Ca^2+^ [mg/L]	Mg^2+^ [mg/L]	K^+^ [mg/L]	Na^+^ [mg/L]
pH	1									
EC [μS/cm]	0.01	1								
TDS [mg/L]	0.01	1	1							
SO_4_^2−^ [mg/L]	0.32	0.41	0.41	1						
Cl^−^ [mg/L]	0.27	0.13	0.13	0.01	1					
HCO_3_^−^ [mg/L]	0.96	0.05	0.05	0.35	0.36	1				
Ca^2+^ [mg/L]	0.82	0.11	0.11	0.31	0.36	0.87	1			
Mg^2+^ [mg/L]	0.73	0.13	0.12	0.28	0.34	0.78	0.92	1		
K^+^ [mg/L]	0.81	0.09	0.09	0.30	0.35	0.85	0.98	0.91	1	
Na^+^ [mg/L]	0.81	0.10	0.10	0.31	0.34	0.86	0.98	0.91	0.99	1

**Table 2:** Descriptive statistics and WQI parameters for groundwater samples.

Parameters					WHO Standards (2011)	Scenario 1 (WQI)		Scenario 2 (WQI)		Scenario 3 (IwQWI)
	Average	Max	Min	STD		Weight (w_i_)	Relative Weight (W_i_)	Weight (w_i_)	Relative Weight (W_i_)	Integreted Weight (W_j_)
Mg^2+^ [mg/L]	14.89	38.4	6.9	7.41	50	2	0.027	-	-	0.019
K^+^ [mg/L]	5.52	12.87	2.73	2.26	12	2	0.027	-	-	0.014
Na^+^ [mg/L]	46.47	107.87	22.77	18.88	200	2	0.027	-	-	0.023
Ca^2+^ [mg/L]	40.81	94.6	20.4	16.54	75	2	0.027	-	-	0.022
SO_4_^2−^ [mg/L]	17.62	28.4	12.5	2.83	250	4	0.055	-	-	0.009
Cl^−^ [mg/L]	24.62	39.5	15.1	5.89	250	3	0.041	-	-	0.011
HCO_3_^−^ [mg/L]	68.98	134.5	21.4	21.98	120	3	0.041	3	0.052	0.017
TDS [mg/L]	625.26	2550	217	361.34	600	4	0.055	4	0.070	0.212
pH	6.88	7.39	6.53	0.15	6.5–8.5	4	0.055	4	0.070	0.004
EC [μS/cm]	1293	5020	455	712	1000	4	0.055	4	0.070	0.405
Mn [mg/L]	0.041	0.17	0.01	0.024	0.4	4	0.055	4	0.070	0.012
Ni [mg/L]	0.033	0.087	0.0009	0.017	0.02	4	0.055	4	0.070	0.032
Fe [mg/L]	0.3	1.22	0.11	0.2	0.3	4	0.055	4	0.070	0.019
NO_3_^−^ [mg/L]	36.22	60.4	21.5	8.56	50	5	0.069	5	0.087	0.012
Cu [mg/L]	0.012	0.035	0.0001	0.007	2	5	0.069	5	0.087	0.027
Al [mg/L]	0.045	0.178	0.002	0.041	0.9	5	0.069	5	0.087	0.054
Zn [mg/L]	0.044	0.129	0.002	0.033	3	5	0.069	5	0.087	0.047
Cr [mg/L]	0.034	0.09	0.01	0.02	0.05	5	0.069	5	0.087	0.018
Pb [mg/L]	0.019	0.06	0.0001	0.016	0.01	5	0.069	5	0.087	0.039
						Ʃ = 72	Ʃ = 1	Ʃ = 57	Ʃ = 1	Ʃ = 1

**Table 3:** Standard dose and guideline values [67].

Element	R*f*D_ingestion_	R*f*D_dermal_	Dermal Permeability Coefficient in Water (Kp)
	(μg/kg/Day)	(μg/kg/Day)	cm/h
Mn	24	0.96	0.001
Ni	20	0.8	0.0002
Fe	700	140	0.001
NO_3_	1600	1600	0.006
Cu	40	8	0.001
Al	1000	200	0.001
Zn	300	60	0.0006
Cr	3	0.075	0.002
Pb	1.4	0.42	0.001

**Table 4:** Number of wells depending on the class of quality.

	WQI Scenario 1		WQI Scenario 2		IwWQI Scenario 3	
	Number of Wells	%	Number of Wells	%	Number of Wells	%
Excellent (<50)	39	48.75	27	33.75	8	10
Good (50–100)	38	47.5	40	50	52	65
Poor (100–200)	3	3.75	13	16.25	17	21.25
Very poor water (200–300)	-	-	-	-	2	2.5
Water is unsuitable for consumption (>300)	-	-	-	-	1	1.25

**Table 5:** Descriptive statistics of effective weights for each parameter.

Parameters	Scenario 1 Effective Weight (E_wi_) (%)				Scenario 2 Effective Weight (E_wi_) (%)				Scenario 3 Effective Weight (E_wi_) (%)			
	Min	Max	Average	STD	Min	Max	Average	STD	Min	Max	Average	STD
Ca^2+^ [mg/L]	0.91	9.66	3.01	1.51	-	-	-	-	0.31	4.60	1.49	0.80
Mg^2+^ [mg/L]	0.58	6.00	1.62	0.93	-	-	-	-	0.20	2.34	0.71	0.44
Na^+^ [mg/L]	0.38	4.13	1.29	0.67	-	-	-	-	0.14	2.04	0.67	0.36
K^+^ [mg/L]	0.76	8.37	2.58	1.35	-	-	-	-	0.17	2.52	0.82	0.45
Cl^−^ [mg/L]	0.31	1.66	0.83	0.32	-	-	-	-	0.03	0.32	0.14	0.05
SO_4_^2−^ [mg/L]	0.34	1.76	0.81	0.32	-	-	-	-	0.02	0.20	0.07	0.03
HCO_3_^−^ [mg/L]	1.06	13.64	4.93	2.41	1.09	14.50	5.22	2.67	0.29	3.99	1.25	0.67
pH	4.58	25.54	11.82	4.85	4.74	27.23	12.53	5.32	0.2	0.15	0.04	0.07
EC [μS/cm]	4.88	21.91	13.26	4.11	5.17	22.70	13.98	4.37	35.69	64.6	54.46	6.22
TDS	3.87	18.55	10.64	3.41	4.10	19.22	11.22	3.62	14.83	27.7	23.66	2.80
Fe [mg/L]	3.36	18.7	9.88	3.52	3.56	19.54	10.40	3.69	0.85	3.85	1.98	0.56
Mn [mg/L]	0.17	2.41	1.06	0.42	0.18	2.56	1.12	0.45	0.03	0.24	0.13	0.03
NO_3_^−^ [mg/L]	3.55	19.50	10.09	3.38	3.64	20.97	10.58	3.72	0.24	2.54	1.10	0.39
Cr [mg/L]	1.69	17.96	8.63	4.41	1.77	18.86	9.00	4.62	0.40	2.38	1.31	0.49
Pb^2+^ [mg/L]	0.03	47.50	19.35	13.07	0.03	48.91	19.96	13.41	0.007	23.35	7.92	6.35
Ni^2+^ [mg/L]	0.07	41.7	14.97	6.99	0.07	42.59	15.67	7.30	0.01	21.83	5.99	3.46
Zn [mg/L]	0.02	0.45	0.16	0.11	0.02	0.46	0.16	0.10	0.005	0.24	0.07	0.05
Al [mg/L]	0.06	2.26	0.53	0.44	0.06	2.33	0.55	0.45	0.02	1.09	0.29	0.26
Cu [mg/L]	0.03	0.25	0.07	0.03	0.01	0.26	0.07	0	0.0002	0.05	0.01	0.01

**Table 6:** HQ values of NO_3_ and metals in Târgovişte Plain.

	HQ Oral				HQ Dermal			
	Average	Max	Min	Stdev	Average	Max	Min	Stdev
Mn	4.93 × 10^−5^	2.02 × 10^−4^	1.19 × 10^−5^	2.90 × 10^−5^	6.16 × 10^−6^	2.53 × 10^−5^	1.49 × 10^−6^	3.63 × 10^−6^
Ni	4.80 × 10^−2^	1.26 × 10^−1^	1.42 × 10^−4^	2.50 × 10^−2^	1.20 × 10^−2^	3.14 × 10^−2^	3.54 × 10^−5^	6.26 × 10^−3^
Fe	1.22 × 10^−2^	4.98 × 10^−2^	4.49 × 10^−3^	8.22 × 10^−3^	3.05 × 10^−4^	1.25 × 10^−3^	1.12 × 10^−4^	2.06 × 10^−4^
NO_3_	6.47 × 10^−1^	1.08	3.84 × 10^−1^	1.53 × 10^−1^	1.94 × 10^−2^	3.24 × 10^−2^	1.15 × 10^−2^	4.60 × 10^−3^
Cu	9.01 × 10^−3^	2.50 × 10^−2^	1.23 × 10^−4^	5.48 × 10^−3^	2.25 × 10^−4^	6.26 × 10^−4^	3.08 × 10^−6^	1.37 × 10^−4^
Al	1.30 × 10^−3^	5.11 × 10^−3^	8.49 × 10^−5^	1.19 × 10^−3^	3.25 × 10^−5^	1.28 × 10^−4^	2.13 × 10^−6^	2.98 × 10^−5^
Zn	4.27 × 10^−3^	1.23 × 10^−2^	2.80 × 10^−4^	3.23 × 10^−3^	6.41 × 10^−5^	1.85 × 10^−4^	4.20 × 10^−6^	4.85 × 10^−5^
Cr	3.24 × 10^−1^	8.57 × 10^−1^	9.52 × 10^−2^	1.90 × 10^−1^	1.30 × 10^−1^	3.43 × 10^−1^	3.81 × 10^−2^	7.62 × 10^−2^
Pb	3.98 × 10^−1^	1.23	3.32 × 10^−4^	3.29 × 10^−1^	6.65 × 10^−3^	2.05 × 10^−2^	5.53 × 10^−6^	5.49 × 10^−3^

**Table 7:** THI and CCR values from Târgovişte Plain.

	THI	HQ _oral_	HQ _dermal_	CCR	CCR		
					Ni	Cr	Pb
average	1.47	1.31	0.16	1.15 × 10^−2^	8.17 × 10^−3^	4.67 × 10^−5^	7.97 × 10^−3^
max	2.97	2.64	0.33	3.92 × 10^−2^	2.14 × 10^−2^	1.25 × 10^−4^	2.46 × 10^−2^
min	0.64	0.59	0.05	1.39 × 10^−5^	2.41 × 10^−5^	1.39 × 10^−5^	6.63 × 10^−6^
stdev	0.56	0.50	0.07	1.01 × 10^−2^	4.26 × 10^−3^	2.80 × 10^−5^	6.58 × 10^−3^

## Data Availability

The data reported in this study are available on request from the first author.
